# A Novel Dopamine Receptor Signaling Unit in Brain: Heterooligomers of D1 And D2 Dopamine Receptors

**DOI:** 10.1100/tsw.2007.223

**Published:** 2007-11-02

**Authors:** Susan R. George, Brian F. O'Dowd

**Affiliations:** ^1^ Department of Pharmacology, University of Toronto, Toronto, Ontario M5S 1A8, Canada; ^2^ Department of Medicine, University of Toronto, Toronto, Ontario M5S 1A8, Canada; ^3^ Centre for Addiction and Mental Health, Toronto, Ontario M5S 1A8, Canada

**Keywords:** dopamine receptor, heterooligomer, D1 dopamine receptor, D2 dopamine receptor, G protein coupled receptor, calcium signal, Gq, oligomerization, striatum, drug addiction, schizophrenia

## Abstract

The ability of G protein coupled receptors to heterooligomerize and create novel signaling complexes has demonstrated the tremendous potential of these receptors to access diverse signaling cascades, as well as to modulate the nature of the transduced signal. In the dopamine receptor field, the existence of a D1-like receptor in brain that activated phospatidylinositol turnover has been shown, but definition of the molecular entity remained elusive. We discovered that the D1 and D2 receptors form a heterooligomer, which on activation of both receptors, coupled to Gq to activate phospholipase C and generate intracellular calcium release. The activation of Gq by the D1-D2 heterooligomer has been shown to occur in cells expressing both receptors, as well as in striatum, distinct from Gs/olf or Gi/o activation by the D1 and D2 receptor homooligomers, respectively. The activation of the D1-D2 receptor heterooligomer in brain led to a calcium signal–mediated increase in phosphorylation of calmodulin kinase lla. The calcium signal rapidly desensitized and the receptors cointernalized after occupancy of either the D1 or D2 binding pocket. Thus, the D1-D2 heterooligomer directly links the action of dopamine to rapid calcium signaling and likely has important effects on dopamine-mediated synaptic plasticity and its functional correlates in brain.

## INTRODUCTION

The analysis of the range of mechanisms of protein-protein interactions has revolutionized the field of G protein coupled receptor (GPCR) biology, and the discovery of direct receptor-receptor interactions has led to the investigation of the varied roles of this process in GPCR function[1,2]. The great strides forward that have occurred in the analysis of the tertiary and quaternary structures of GPCRs through biophysical, protein crystallography, and atomic force microscopy methods[3,4,5,6,7,8] have aided the understanding and interpretation of functional studies that revealed the presence of receptor oligomers. The realization of the significance of GPCR homo- and heterooligomerization[9] as these processes are being further analyzed has added another degree of complexity to the understanding of this important class of receptors.

One of the most exciting aspects of GPCR oligomerization has been the observation that heterooligomerization gives rise to receptor complexes with properties that are completely distinct from that of their constituent receptors. This has been shown to include novel pharmacology[10,11], masking the effect of one component[12,13] or enhancing the efficacy of one of the receptors[14,15], to name a few. Some of the functional effects mediated by GPCR heterooligomers were discovered on the basis of physiological findings for which activation of the known GPCRs did not provide a mechanism.

## THE SEARCH FOR A Gq-COUPLED D1 RECEPTOR

In the dopamine receptor field, it was the quest for the Gq-coupled D1-like receptor that led us to identify the D1-D2 receptor heterooligomer. Although the cloning efforts of our Toronto group and others identified five separate dopamine receptors, which were categorized as D1-like or D2-like based on their pharmacological properties, gene and protein structure, G protein linkage, and ability to activate or attenuate adenylyl cyclase activity, these receptors provided no readily available identification of a D1-like receptor coupled to Gq in striatum. The cloned D1 receptor, when expressed in heterologous cells, such as Cos7[16], HEK[17], CHO, or BHK[18] cells, did not activate a phospholipase C–dependent calcium signal, whereas in brain tissue[19,20] or *Xenopus* oocytes injected with striatal mRNA[21], there was evidence for a D1 receptor activated phospholipase C–mediated increase in phosphatidylinositol turnover. Clearly, this discrepancy suggested the presence of some endogenous mechanism in native tissues that was absent in all of the heterologous expression systems. A definitive pharmacological profile for this "D1 receptor" was not established and many studies used relatively high concentrations of drugs, calling into question the agonist specificity of the observed effects. This led us to consider the possibility that the D1 agonist stimulated phospholipase C and phosphatidylinositol turnover may have resulted from the coincident activation of another GPCR in brain.

Lending further support to this notion was the long-accumulated evidence from a variety of sources indicating that certain dopamine D1 and D2 receptor mediated effects and behaviors were synergistic and resulted from the activation of both receptors. Since the molecular bases of D1 and D2 receptor modulation of adenylyl cyclase activity were inherently opposite, alternate mechanisms likely mediated these effects. The identity of the dopamine receptors that could activate inositol triphosphate production appeared to be distinct, because the ability of certain drugs to activate this pathway did not correlate with their ability to activate adenylyl cyclase[22], indicating that the Gq-coupled dopamine D1 receptor was distinct from the Gs/olf-coupled D1 or Gi/o-coupled D2 dopamine receptors. Since we postulated that the factor absent in D1-expressing heterologous cells, but present in striatal tissue, might be another GPCR, we considered receptors present in striatum that could be activated by high concentrations of D1 receptor agonists, such as the D2 dopamine receptor, since the density of D5 receptors in striatum is low. Since GPCRs have been shown to heterooligomerize[1], we investigated this possibility and demonstrated that D1 and D2 receptors were present within a certain proportion of striatal neurons using selective antibodies that were specific without cross-reactivity to any of the other dopamine receptors[17]. The ability to coimmunoprecipitate the D1 receptor along with the D2 receptor and vice versa from cells coexpressing both receptors, as well as from striatal tissue, indicated that the receptors were expressed within the same signaling complex in cells and in brain. Furthermore, biophysical analytic methods to evaluate energy transfer using cell surface FRET[23] or BRET throughout the cell (unpublished) reveal that the D1 and D2 receptors exist together in close proximity of 50–100 Å on the cell surface and within the endoplasmic reticulum.

## DOPAMINE ACTIVATES A CALCIUM SIGNAL THROUGH THE D1-D2 HETEROOLIGOMER

Since the classical signaling pathways activated by D1 and D2 dopamine receptors involve the stimulation or attenuation of adenylyl cyclase–mediated generation of cyclic AMP, it appeared unlikely that the D1-D2 heterooligomer would function through a cyclic AMP–related mechanism. Therefore, we investigated other second messenger pathways and demonstrated that activation of both receptors within the D1-D2 heterooligomer triggered intracellular calcium release through a Gq-mediated phospholipase C–dependent pathway[17]. The calcium signal was attenuated by an antagonist of either the D1 or D2 receptor, indicating the necessity for activation of both constituents of the heterooligomer to elicit the signal. Further characterization of the D1-D2–activated calcium signal has delineated the cascade to include activation of the inositol triphosphate receptor and lack of a role for extracellular calcium influx[24]. Roles for other intracellular pathways that could trigger calcium release have been excluded through the use of inhibitors of multiple components of these signaling cascades.

## ACTIVATION OF THE D1-D2 HETEROOLIGOMER IN STRIATUM

The activation of the D1-D2 heterooligomer by dopamine agonists in rat and mouse striatum induced rapid activation of Gq/11, as indicated by direct GTP$\gamma^{35}$S incorporation into the G protein[24]. This was absent in both D1 or D2 receptor gene-deleted animals, underscoring the need for involvement of both receptors for the Gq activation effect. Furthermore, just as documented in the heterologous cell lines, treatment with either the D1 or D2 receptor antagonist abolished the effect of agonist activation, emphasizing the necessary participation of both of the constituent receptors in generating the functional response. Thus, the presence and activation of both D1 and D2 receptors within the heterooligomeric complex appears necessary for activation of Gq in striatum by dopamine agonists.

Generation of a dopamine-triggered calcium signal through D1-D2 heteromer activation will lead to the initiation of several signaling cascades subsequently. One of the more immediate consequences of this would be activation of calmodulin and calmodulin kinase (CaMK). Since CaMKllα is the most abundant isoform of CaMK relevant for synaptic functions, we examined the effect of the calcium signal activation on CaMKllα function. Within 5–10 min following concurrent administration of a D1 and D2 agonist given intraperitoneally to mice and rats, we found marked increases of phosphorylated CaMKllα in neurons of nucleus accumbens shell and certain areas of caudate nucleus. This increase could be blocked by preadministration of the D1 antagonist SCH 23390 or the D2 antagonist raclopride, and did not occur in D1 or D2 gene-deleted mice[24]. Blocking NMDA receptors using MK-801 had only a minor effect on the phospho-CaMKllα levels generated following D1-D2 heteromer activation.

## DEFINING A NOVEL PHARMACOLOGY FOR THE D1-D2 HETEROOLIGOMER

Our early studies examining dopamine or the commonly used D1 or D2 dopamine agonists (e.g., SKF 81297 or quinpirole) found no differences between the ligand binding pocket of the D1 or D2 receptor within the D1-D2 heterooligomer with that within their respective homooligomers[23]. The D1 and D2 receptor selective antagonists also had unaltered binding characteristics whether binding to the receptors within the homooligomer or heterooligomer. However, since the G protein specificity of the D1-D2 heterooligomer differed from that of the D1 and D2 homooligomers, we reasoned that there could be significant alterations to the intracellular domains of the receptors that enabled coupling to a different G protein and thus postulated that there may also be alterations to the ligand binding pockets. We therefore screened a number of diverse dopaminergic agonists to detect these differences. Using a series of SKF compounds, we identified several interesting pharmacological profiles with differential specificity for the receptor homo- and heterooligomers. For instance, the commonly used D1 agonist SKF 81297 had potent affinity for the D1 receptor in D1-D2 and D1-D1 complexes, with $K_D$ values in the nanomolar range. This agonist robustly stimulated Gs-mediated adenylyl cyclase activity as well as Gq-mediated intracellular calcium release. In contrast, two other SKF compounds of interest include SKF 83959 and SKF 83822, both of which had indistinguishable high binding affinity for the D1 receptor in D1-D2 or D1-D1 complexes. However, we showed that these agonists had very specific functional effects, since SKF 83959 robustly stimulated a calcium signal and did not activate adenylyl cyclase, whereas SKF 83822 robustly stimulated adenylyl cyclase with no effect to stimulate calcium release. Thus, these agonists had selective actions to activate the D1-D2 heterooligomer and the D1-D1 homooligomer, respectively[24].

Since many of our other studies *in vitro* and *in vivo* had indicated the necessity of occupying and activating both D1 and D2 receptors, it appeared paradoxical that certain SKF "D1 agonists" could generate a calcium signal by their sole administration. We investigated this by examining the binding pockets within each receptor and determined that these D1 agonists could activate Gq/11 by acting as a full agonist at the D1 receptor and a partial agonist at the D2 receptor within the D1-D2 heteromeric complex[24]. This was verified experimentally by two strategies. First, comparison of the peak heights of the calcium signal generated by the SKF compounds revealed it was 60–75% of that generated by dopamine, and second, coadministration of the D2 agonist quinpirole with the SKF agonists increased calcium release maximally, to the same extent as with dopamine[24,25]. Furthermore, the use of pertussis toxin (PTX) to uncouple Gi/o proteins from D2 and render the D2 homooligomer of low affinity to agonists revealed that SKF 81297 and SKF 83959 bound to a PTX-insensitive D2 receptor with high affinity, which represents the D2 receptor linked to the PTX-insensitive Gq protein. These agonists detected a high-affinity PTX-resistant D2 site that was documented in rat and mouse striatum, as well as in cells expressing D1-D2 complexes, but was absent in cells expressing only D1 or D2 receptors or in D1 gene-deleted animals. These results indicate that the presently accepted pharmacological profile for dopamine agonists will need to be re-examined and revised to include the rank orders at each of the distinct D1-D2, D1-D1, and D2-D2 receptor signaling complexes.

## TURNING OFF THE CALCIUM SIGNAL

The peak height of the calcium signal activated by the D1-D2 heterooligomer occurs within a few seconds of agonist administration and was found to desensitize within minutes[25]. Interestingly, agonist occupancy of either ligand binding pocket within the heterooligomer resulted in desensitization of the calcium signal. Pretreatment with either a D1 or a D2 agonist induced desensitization. Exclusive occupancy of the D1 binding pocket was ensured by coadministration of a D2 antagonist. Following this pretreatment, the dopamine-stimulated calcium signal was blunted and desensitized. Similarly, pretreatment with a D2 agonist with the D1 binding pocket occluded by an antagonist also resulted in desensitization to subsequent dopamine activation. However, the kinetics of desensitization was different based on whether the D1 or D2 receptor was occupied by agonist. Desensitization by D1 receptor occupancy was most rapid, occurring with a half-life of 2 min, whereas desensitization by D2 receptor occupancy occurred with a half-life of 10 min[25]. The mechanism of desensitization involved the noncatalytic actions of GPCR kinases 2 and 3. The heteromeric complexes underwent internalization documented 20 min after agonist activation. The removal of the complexes off the cell surface occurred following occupancy of either the D1 or D2 binding pocket[25].

## SUMMARY AND SIGNIFICANCE

We have identified a novel mechanism through which dopamine can activate the fast-signaling intracellular calcium release machinery in brain through the activation of D1-D2 heterooligomers. Calcium signaling has important roles in many aspects of neuronal function, and activation of the calcium signaling cascade will enable dopamine neurotransmission to have rapid and direct effects on synaptic plasticity and long-term changes as a consequence. The delineation of the functional responses mediated by the activation of the D1-D2 dopamine receptor heterooligomer will dissect out its potential roles in cognition, learning, and memory, as well as in the pathological processes related to disorders such as schizophrenia and drug addiction.

Stemming from our realization that oligomerization is an important characteristic of the structure and function of GPCRs, heterooligomerization presents yet another novel facet of these receptors, increasing the complexity of the mechanisms of action of neurotransmitters, established drugs, and the molecular models of diseases that have been studied for many years. Increased understanding of the full implications of these receptor-receptor interactions among GPCRs will undoubtedly yield significant insights into their function and the behaviors and clinical disorders that are mediated by these receptors.

## ACKNOWLEDGMENTS

This work was supported by grants from the National Institute on Drug Abuse and the Canadian Institutes for Health Research. SRG holds a Tier 1 Canada Research Chair in Molecular Neuroscience.
